# A computational study of stochastic mechanisms in dendritic calcium spike generation

**DOI:** 10.1186/1471-2202-13-S1-P152

**Published:** 2012-07-16

**Authors:** Haroon Anwar, Iain Hepburn, Erik De Schutter

**Affiliations:** 1 Computational Neuroscience Unit, Okinawa Institute of Science and Technology, Okinawa 904-0411, Japan; 2 Theoretical Neurobiology, University of Antwerp, B-2610 Antwerpen, Belgium

## 

Neuronal activity is largely influenced by voltage-dependent and calcium-dependent ion channels, and their interaction with calcium related mechanisms present in and around the complex cellular morphology. In the past, people have focused on stochasticity of voltage-gated ion channels to study its effect on neuronal excitability [1-4] but have ignored the intracellular aspects, in particular calcium dynamics. It is our aim to clarify the role of stochasticity of intracellular calcium dynamics in modulating neuronal output. Intracellular calcium dynamics in neuronal systems significantly control their firing pattern, such as a calcium spike. A calcium spike is generated by interaction of voltage gated Ca^2+^ channels and Ca^2+^-activated K^+^ channels, where the interaction is mediated through intracellular calcium mechanisms. A significant proportion of calcium entering through voltage-gated calcium channels binds to buffers, diffuses away and is extruded. Only a limited amount of calcium binds to Ca^2+^-activated K^+^ channels to conform it to conducting states. These complex interactions take place in and around complex cellular morphology, where stochastic interaction between diffusing molecules and surface bound molecules, stochastic transitions between ion channel conformations and variability in molecular arrangement may have a significant effect on neuronal excitability.

We studied the stochastic behavior of dendritic calcium spikes in Purkinje neurons. In our study, we used a model for dendritic calcium spikes, which included P- and T- type Ca^2+^ channels, BK- and SK- type Ca^2+^-activated K^+^ channels, parvalbumin and calbindin as calcium buffers, pumps, diffusion of Ca^2+^ molecules, diffusion of free buffers and diffusion of Ca^2+^ bound buffers. Details about the ion channel kinetics, buffer kinetics and diffusion rate constants used in this study can be found in Anwar et al. 2010 [5] .We ran all simulations in STEPS [6], which supports stochastic and deterministic molecular simulations alongside accurate computation of the electrical behavior of the cellular region, all within complex 3D morphologies. Therefore we could run a series of stochastic, deterministic and hybrid simulations, investigate the different sources of noise individually and together, and compare to deterministic solutions.

By comparing the sources of noise in this system in absolute and relative terms at different lengths of dendritic section we demonstrate the significance of the different contributing factors to stochasticity in the system, including calcium dynamics and ion channel gating, on a variety of spatial scales.

## References

[B1] Cannon RC O’Donnell C Nolan MF Stochastic ion channel gating in dendritic neurons: morphology dependence and probabilistic synaptic activation of dendritic spikes PLoS Comp Biol 2010 6 e1000886 10.1371/journal.pcbi.1000886 PMC2920836 20711353

[B2] Careli PV Reyes MB Sartorelli JC Pinto RD Whole cell stochastic model reproduces the irregularities found in the membrane potential of bursting neurons J Neurophysiol 2005 94 1169 1179 10.1152/jn.00070.2005 15800078

[B3] Dudmann JT Nolan MF Stochastically gating ion channels enable patterned spike firing through activity-dependent modulation of spike probability PLoS Comp Biol 2009 5 e1000290 10.1371/journal.pcbi.1000290 PMC2631146 19214199

[B4] Schneidman E Freedman B Segev I Ion channel stochasticity may be critical in determining the reliability and precision of spike timing Neural Comput 1998 10 1679 1703 10.1162/089976698300017089 9744892

[B5] Anwar H Hong S De Schutter E Controlling Ca(2+)-Activated K (+) Channels with Models of Ca (2+) Buffering in Purkinje Cells Cerebellum 2010 DOI: 10.1007/s12311-010-0224-3 10.1007/s12311-010-0224-3 PMC3411306 20981513

[B6] STEPS: STochastic Engine for Pathway Simulation http://steps.sourceforge.net/

